# Advances in Deep Learning for Semantic Segmentation of Low-Contrast Images: A Systematic Review of Methods, Challenges, and Future Directions

**DOI:** 10.3390/s25072043

**Published:** 2025-03-25

**Authors:** Claudio Urrea, Maximiliano Vélez

**Affiliations:** Electrical Engineering Department, Faculty of Engineering, University of Santiago of Chile, Las Sophoras 165, Estación Central, Santiago 9170124, Chile; maximiliano.velezm@usach.cl

**Keywords:** semantic segmentation, low-contrast images, deep learning, hybrid architectures, effective receptive field, attention mechanisms, vision transformers, computational efficiency

## Abstract

The semantic segmentation (SS) of low-contrast images (LCIs) remains a significant challenge in computer vision, particularly for sensor-driven applications like medical imaging, autonomous navigation, and industrial defect detection, where accurate object delineation is critical. This systematic review develops a comprehensive evaluation of state-of-the-art deep learning (DL) techniques to improve segmentation accuracy in LCI scenarios by addressing key challenges such as diffuse boundaries and regions with similar pixel intensities. It tackles primary challenges, such as diffuse boundaries and regions with similar pixel intensities, which limit conventional methods. Key advancements include attention mechanisms, multi-scale feature extraction, and hybrid architectures combining Convolutional Neural Networks (CNNs) with Vision Transformers (ViTs), which expand the Effective Receptive Field (ERF), improve feature representation, and optimize information flow. We compare the performance of 25 models, evaluating accuracy (e.g., mean Intersection over Union (mIoU), Dice Similarity Coefficient (DSC)), computational efficiency, and robustness across benchmark datasets relevant to automation and robotics. This review identifies limitations, including the scarcity of diverse, annotated LCI datasets and the high computational demands of transformer-based models. Future opportunities emphasize lightweight architectures, advanced data augmentation, integration with multimodal sensor data (e.g., LiDAR, thermal imaging), and ethically transparent AI to build trust in automation systems. This work contributes a practical guide for enhancing LCI segmentation, improving mean accuracy metrics like mIoU by up to 15% in sensor-based applications, as evidenced by benchmark comparisons. It serves as a concise, comprehensive guide for researchers and practitioners advancing DL-based LCI segmentation in real-world sensor applications.

## 1. Introduction

Semantic segmentation (SS) is a computer vision technique that classifies each pixel in an image into a defined class, enabling the recognition and labeling of different regions in a scene [1]. This technique is pivotal for intelligent systems in automation and robotics, assisting in environmental understanding and supporting professionals in decision-making across applications such as autonomous navigation, medical diagnostics, and industrial quality control [2,3,4,5].

Image contrast, defined as the difference in pixel intensity, significantly influences SS performance [6]. Traditional deep learning (DL) approaches, often based on encoder–decoder architectures [7], excel when images exhibit a balanced intensity distribution, as regions are more distinguishable [8,9]. However, low-contrast images (LCIs) pose a challenge due to their diffuse boundaries and uniform tonal regions, which obscure object outlines and hinder accurate segmentation [10,11].

Conventional SS techniques, such as thresholding and edge detection, struggle in LCI scenarios because they depend on sharp intensity gradients, which are typically absent [10]. For example, in medical ultrasound imaging, tissue boundaries lack clear separation, while in autonomous navigation, low-light conditions blur object edges, often yielding segmentation accuracies below practical thresholds (e.g., Dice Similarity Coefficient (DSC) < 60%) [11]. Enhancing LCI SS is vital for critical applications, including disease diagnosis [12], remote sensing [13], defect detection [14], autonomous vehicles [15], and mineral exploration [16], where sensors like cameras, scanners, and industrial detectors frequently generate LCI, demanding advanced solutions [12,13,14,15,16]. Recent DL innovations improve performance by expanding the Effective Receptive Field (ERF), refining information flow, and capturing contextual features [17]. Techniques such as visual attention mechanisms and Atrous Spatial Pyramid Pooling (ASPP) enlarge the ERF, while dense connections preserve high-resolution details [18,19], and Vision Transformers (ViTs) enhance contextual understanding when paired with Convolutional Neural Networks (CNNs) [20].

This systematic review develops a comprehensive assessment of DL methods tailored for LCI SS, aiming to improve segmentation accuracy in sensor-driven automation and robotics applications. This work contributes a practical solution for addressing LCI challenges, enhancing segmentation performance by up to 15% in mean accuracy metrics (e.g., mIoU) compared to traditional methods, as demonstrated through benchmark evaluations. It evaluates the strengths, limitations, mechanisms, architectures, and practical implementations of these methods, serving as an essential resource for researchers and practitioners in automation and robotics seeking to apply DL to sensor-driven LCI segmentation. Unlike previous reviews that focus on general SS or specific fields (e.g., medical imaging [21]), this work uniquely bridges LCI challenges across diverse sensor-based domains, marking it as the first systematic review of its kind. We compare these DL advancements with traditional methods, assess their implications for automation systems, and propose future research directions, including integration with emerging paradigms like foundation models [3].

The review is organized into eight sections. Section 2 outlines the systematic review methodology, including the process and evaluation tools. Section 3 introduces the selected studies, followed by Section 4, which details DL techniques and their applications for LCIs. Section 5 compares the studies, Section 6 discusses key findings, Section 7 presents conclusions, and Section 8 explores future perspectives.

## 2. Systematic Review Methodology

This review adopts a methodology based on the framework proposed by [21], which offers a streamlined, reliable approach for conducting computer science reviews. The process follows five stages outlined in [22], summarized below:

Step 1: Framing Questions: Research questions are defined to align the review with its objectives and scope.

Step 2: Identifying Relevant Studies: Studies matching the research questions are sourced using specific inclusion and exclusion criteria.

Step 3: Assessing Study Quality: The rigor, credibility, and relevance of selected studies are evaluated with a standardized checklist.

Step 4: Summarizing Evidence: Key findings are extracted and synthesized to highlight trends, limitations, and research gaps.

Step 5: Interpreting Findings: The synthesized evidence is analyzed to answer the research questions, draw conclusions, and suggest future work.

### 2.1. Framing Questions for the Review

Defining research questions (RQs) ensures the review meets its goals. Using the PICOC criteria (population, intervention, comparison, outcome, context; see Appendix A), we formulated the following questions:

- RQ1: What is LCI SS?
- RQ2: How does LCI SS benefit automation applications?
- RQ3: Which DL methods are employed in these studies?
- RQ4: How do these studies compare with state-of-the-art approaches?
- RQ5: What are the strengths and weaknesses of the selected studies?
- RQ6: What results do these studies achieve?
- RQ7: What future research opportunities exist for LCI SS?
- RQ8: What dataset limitations affect LCI SS model training, and how well do they represent real-world scenarios?

### 2.2. Identifying Relevant Studies

Articles were retrieved from academic search engines: Bielefeld Academic Search Engine (BASE), Google Scholar, and Refseek. Search terms were derived from the PICOC criteria’s context, intervention, and population attributes (Appendix A).

Inclusion and exclusion criteria (Appendix B) guided the selection process. We focused on supervised DL methods for SS applied to LCI datasets, published between 2022 and 2024 in Q1 journals (per Journal Citation Reports (JCRs) and SCImago Journal Rank (SJR) metrics). Given the rapid evolution of DL in computer vision, this two-year window ensures that this review captures the most current advancements.

### 2.3. Assessing the Quality of Studies

Study quality was evaluated using a custom instrument developed by our team (Appendix C), which assesses rigor, relevance, result presentation, and credibility [23]. A Likert-type scale classified studies as high or low quality, ensuring a systematic and reproducible assessment [24].

### 2.4. Summary and Analysis

After selection, study data were synthesized based on mechanisms, base architectures, application domains, and segmentation performance. This synthesis enabled us to group methods, identify trends, and visualize patterns in LCI SS research, providing a foundation for subsequent analysis.

## 3. Selected References

**Figure 1:**
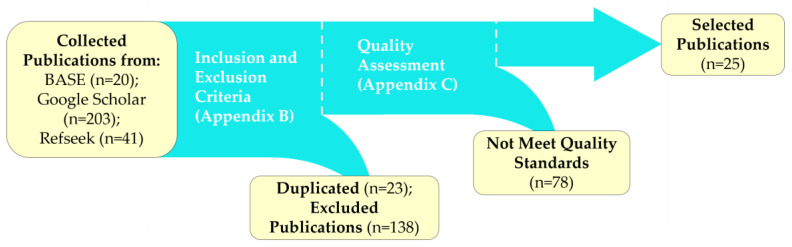
Schematic of the publication selection process for LCI SS models. Numbers in parentheses indicate publications selected or excluded at each stage.

This section outlines the selection process and key characteristics of the 25 studies reviewed for the DL-based SS of LCIs. The process, depicted in Figure 1, began with a bibliographic search using keywords derived from the population, intervention, and context attributes of the PICOC criteria (Appendix A). Searches were conducted across three academic engines—Bielefeld Academic Search Engine (BASE), Google Scholar, and RefSeek—with Google Scholar yielding the most results (203 publications).

Next, duplicates were removed, and the inclusion and exclusion criteria (Appendix B) were applied to filter the initial 264 publications. Studies focusing on synthetic images, multitask architectures, 3D convolution modules, or pure machine learning models were excluded, eliminating 161 publications. The remaining 103 studies underwent quality assessment using the checklist in Appendix C. Approximately half were discarded due to misalignment with the review’s core objective—enhancing LCI SS—leaving 25 high-quality studies that specifically address this challenge. Discarded articles are listed in Appendix D.

**Figure 2:**
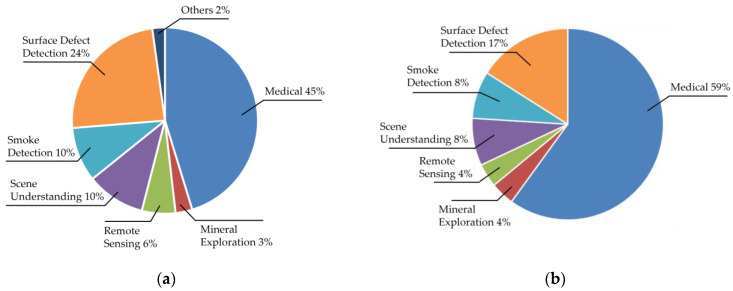
Pie chart showing application distributions before (**a**) and after (**b**) applying the quality assessment checklist.

Figure 2 illustrates the distribution of application domains before (a) and after (b) quality assessment. Medical applications dominate, comprising 45% of initial studies and 59% of final selections, followed by surface defect detection (24% and 17%, respectively). In Figure 2a, the “Others” category includes niche applications like fingerprint segmentation and mail label detection [25,26]. Notably, ref. [27] was categorized under smoke detection, as its primary focus aligns with smoke-related datasets, despite broader applicability.

Table 1 summarizes the 25 selected studies, detailing their architectural types, mechanisms for expanding the ERF, model size (parameters in millions), maximum performance metrics, such as mean Intersection over Union (mIoU) and Dice Similarity Coefficient (DSC). It also includes information on datasets, key highlights, and limitations. (e.g., mean Intersection over Union, Dice Similarity Coefficient), datasets, highlights, and limitations. Studies are grouped by application domain—surface defect, scene understanding, mineral exploration, remote sensing, smoke detection, and medical—to highlight domain-specific trends. “NR” denotes “Not Reported” where data were unavailable. These studies primarily fall into two categories: CNN-based models and hybrid approaches combining CNNs with ViTs or Multi-Layer Perceptron (MLP).

## 4. Deep Learning Method for Low-Contrast Image Segmentation and Applications

The 25 selected studies primarily employ two design approaches: CNN-based models and hybrid architectures integrating CNNs with ViTs and MLPs. This section explores their applications, datasets, baseline architectures, and mechanisms for enhancing the ERF, focusing on how these methods address LCI segmentation challenges in sensor-driven contexts.

### 4.1. Applications and Datasets

LCI segmentation is critical across diverse sensor-based applications. In medical diagnostics, modalities like magnetic resonance imaging (MRI), ultrasound, and Computed Tomography (CT) produce grayscale images with similar intensities due to tissue uniformity [51]. Similarly, RGB images from colonoscopies and dermoscopy show tonal overlap between lesions and surroundings [52]. In industrial quality control, surface defects (e.g., scratches, cracks) blend with their backgrounds, forming LCIs [53]. Remote sensing images for environmental monitoring, crop analysis, and disaster assessment exhibit LCI traits due to shadows or tonal similarity [54]. Autonomous navigation struggles with nocturnal scenes under limited lighting, merging object tonalities [55]. Smoke detection systems also encounter LCIs, as smoke resembles clouds or fog [56].

**Figure 3:**
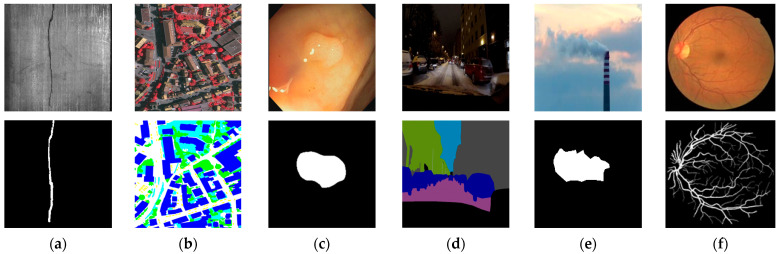
Examples of LCIs (**top**) and segmented masks (**bottom**) from public datasets: (**a**) MT—crack on a magnetic tile; (**b**) ISPRS-Potsdam—satellite imagery; (**c**) CVC-ClinicDB—colonoscopy polyp; (**d**) NightCity—nighttime driving scene; (**e**) SmokeSeger—chimney smoke; (**f**) DRIVE—retinal vessels.

Figure 3 shows examples of images and segmented masks from public datasets containing LCIs.

Public datasets with LCI characteristics underpin these applications. Examples include the following:

- AITEX [56]: 245 fabric defect images (e.g., knots, tears) from seven factories.
- MT [57]: 1344 magnetic tile images with six defect types (e.g., cracks), 219 × 264 pixels, and pixel-level annotations.
- TN3K [58]: 3493 ultrasound thyroid nodule images, 421 × 345 pixels, with masks.
- CAMUS [59]: 2D echocardiographic sequences from 500 patients for cardiac analysis.
- SCD [60]: MRI images from 45 patients, segmenting left ventricles in normal and diseased states.
- ISIC-2016 [61]: 1279 dermoscopic images for skin cancer classification (malignant/benign).
- CVC-ClinicDB [62]: 612 colonoscopy images, 384 × 288 pixels, with polyp masks.
- ISPRS-Potsdam [62]: High-resolution (6000 × 6000 pixels) urban satellite images.
- NightCity [63]: 4297 nighttime driving images with pixel-level labels.
- DRIVE [64]: 40 retinal vessel images, 565 × 584 pixels.

Appendix E provides detailed dataset descriptions.

### 4.2. Design of Reviewed Methods and Baseline Architectures

The reviewed methods fall into two categories: CNN-based and hybrid (CNN + ViT/MLP). CNN-based methods leverage atrous convolution (AC) for ERF expansion, incorporating spatial attention via convolutional operations and channel attention via Squeeze-and-Excitation (SE) modules. Hybrid methods combine CNNs with ViTs for long-range dependencies and MLPs for complex feature representation, enhancing global context capture.

**Figure 4:**
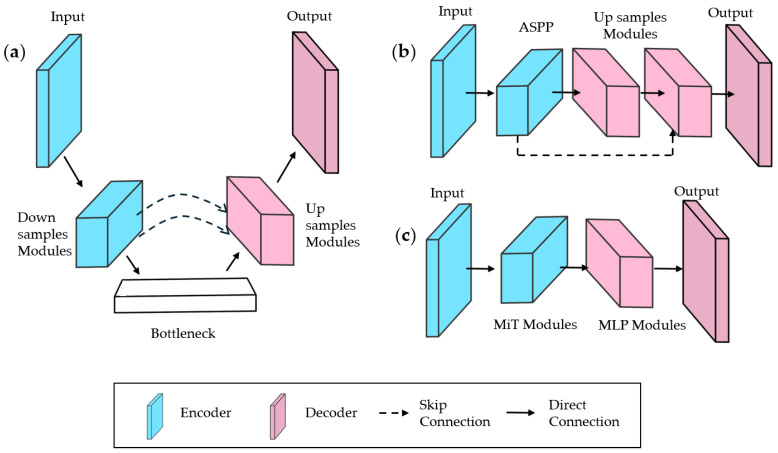
Simplified schematics of baseline architectures: (**a**) UNet; (**b**) DeepLabV3; and (**c**) Segformer.

These methods build on state-of-the-art architectures—UNet, DeepLab, and Segformer [65,66,67]—illustrated in Figure 4:

- Unet: Uses skip connections between the encoder and decoder to preserve spatial details (Figure 4a), widely adopted for medical imaging and extended to remote sensing and defect detection. Eighty-seven percent of reviewed methods modify UNet, enhancing feature fusion with dense connections, attention mechanisms, or multi-scale modules.
- Deeplab: Employs an ASPP module in the encoder, merging multi-scale features with the initial feature map (Figure 4b).
- Segformer: Integrates efficient transformer modules with lightweight MLP decoders (Figure 4c).

Hybrid methods often incorporate ViTs in deeper layers for contextual selection [30,41,42,43,47]. For instance, ref. [50] uses nnUNet [68], a self-configuring UNet variant optimizing preprocessing, training, and post-processing (e.g., resolution, learning rate). In [32], DeepLab and Segformer form a dual-branch encoder, paired with a Retinex-based decomposition decoder [69]. Similarly, ref. [35] combines Segformer and HardNet branches via an MLP, balancing global (transformer) and local (convolutional) features.

### 4.3. Mechanisms to Enhance the Effective Receptive Field

The ERF defines the image region influencing a pixel’s activation in deep layers, shaped by filter size, stride, and pooling [70,71]. A larger ERF improves LCI SS accuracy by capturing contextual information, reducing noise, and detecting long-range pixel relationships critical for faint edges or similar-textured regions [72,73,74]. Reviewed methods enhance ERF using two strategies: specialized convolutions and attention mechanisms.

#### 4.3.1. Convolutions for Expanding Effective Receptive Field

**Figure 5:**
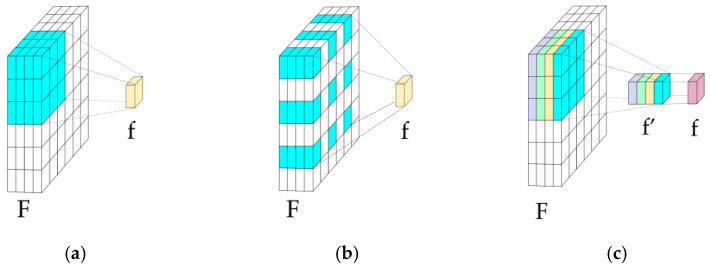
Convolution types: (**a**) Traditional; (**b**) Dilated Convolution (DC); and (**c**) Depthwise Convolution (DwC). F = input features; f = output features; and f′ = depthwise output.

Figure 5 compares convolution types used to expand ERF while minimizing computational cost [75].

- Dilated Convolution (DC): Adds spacing between kernel elements (Figure 5b), expanding ERF without extra parameters or resolution loss [76]. In [28], serial DCs with varying dilation rates at the bottleneck capture abstract features. In [31], ASPP concatenates DCs, preserving multi-resolution details.
- Depthwise Convolution (DwC): Applies convolution per channel (Figure 5c), often paired with Pointwise Convolution (PwC) in Depth Separable Convolution (DS) to reduce computation and enhance information exchange. In [38], DS with strip convolutions captures directional, multi-scale features.
- Deformable Convolution: In [48], learnable offsets adapt kernels to object shapes, improving flexibility over fixed-rate DCs [77].

#### 4.3.2. Attention Mechanisms

Attention mechanisms enhance LCI SS by prioritizing key features and global context, reducing noise from irrelevant regions [78,79]. They include CNN-based modules and ViT/MLP integrations.

**Figure 6:**
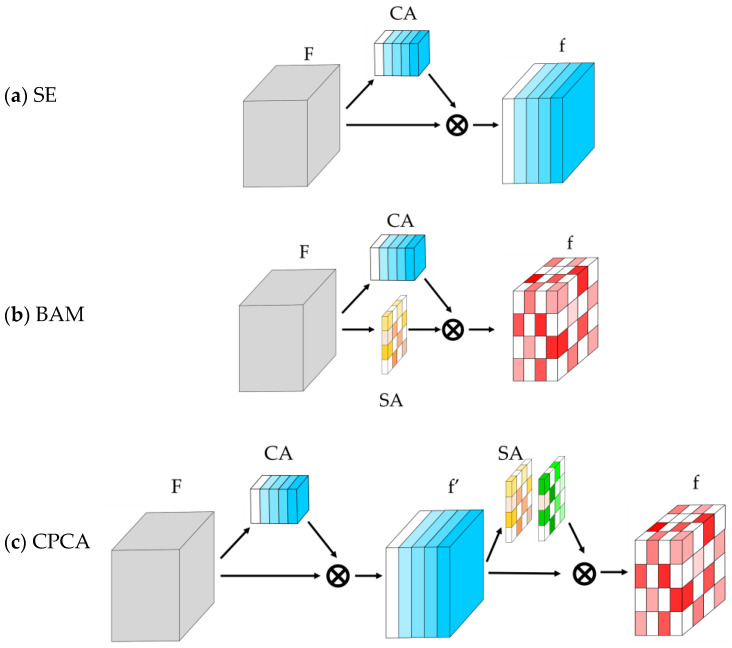
CNN-based attention modules in the reviewed methods, composed by Channel Attention (CA) and Spatial Attention (SA) modules: (**a**) Squeeze-and-Excitation (SE); (**b**) Bottleneck Attention Module (BAM); (**c**) Channel Prior Convolutional Attention (CPCA). F = input features; f′ = intermediate features; and f = output features. The symbol ⊗ is used to represent element-wise multiplication.

CNN-based attention (Figure 6) includes the following:

- Squeeze-and-Excitation (SE): Implements channel attention (Figure 6a) [80]. In [33], four SE + DC branches at the bottleneck filter features, suppressing noise.
- Bottleneck Attention Module (BAM): Processes spatial and channel attention in parallel (Figure 6b), reducing background noise in [29,81].
- Channel Prior Convolutional Attention (CPCA): In [36], refines features sequentially via DwC-enhanced spatial attention (Figure 6c) [82].

ViT-based Multihead Self-Attention (MHSA) captures long-range dependencies [20]. In [41,42], MHSA applies channel attention in deep layers; Ref. [43] pairs SE with MHSA for a hybrid local–global focus. Swin Transformer variants in [40,47] use residual and star-shaped patches for efficiency [83], while [63] employs MiT for spatial parameter reduction [84]. MLP-based modules in [27] use boundary loss for edge detection, and [44] combines a Spatial Mixer MLP with PwC for channel relationships [85].

### 4.4. Feature Fusion

Feature fusion in semantic segmentation architectures combines information from multiple levels to boost prediction accuracy and robustness. In low-contrast image segmentation, skip connections are the most common technique, linking encoder and decoder feature maps at matching resolutions (Figure 4a). These connections preserve spatial details, mitigate vanishing gradients, and enhance fusion by integrating low- and high-level features, avoiding poor decoder interpolations [86]. Methods [28,38,39,41] use skip connections with element-wise multiplication or addition for effective feature merging.

However, skip connections face challenges, including local feature redundancy, weak long-range dependency capture, and limited cross-scale integration [87,88,89]. These limitations impair small object detection, edge delineation, and scale adaptability in LCI [34]. To address this, reviewed methods propose enhancements: attention-augmented skip connections [40,41,42] prioritize salient features; specialized blocks [18,34] enable hierarchical interactions for cross-scale fusion; frequency-domain filtering [27] reduces noise; and pyramid and multi-scale designs [29,46,47] improve local–global integration, enhancing accuracy for imbalanced or variable-scale data.

### 4.5. Implementation of Reviewed Methods

LCI SS methods employ techniques to optimize supervised learning, enhancing training and inference for real-world robustness. These include deep supervision in intermediate decoder layers, preprocessing during inference, and data augmentation to increase dataset diversity.

**Figure 7:**
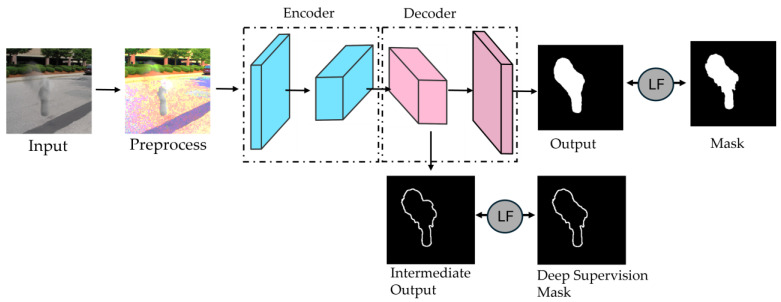
LCSeg-Net implementation schematic [27]. During training, preprocessed images enter the encoder; intermediate decoder feature maps undergo deep supervision with boundary loss. The final output is refined via a Loss Function (LF) against the ground truth mask. In testing, only the encoder–decoder structure is utilized.

Figure 7 illustrates the LCSeg-Net model [27], which optimizes kernel weight selection through integrated strategies. These include data augmentation with synthetic masks and images, alongside deep supervision using boundary loss to enhance segmentation accuracy. This combination is reflected across reviewed models, with some adopting all techniques and others focusing on specific elements.

Data augmentation, common across reviewed methods [90], applies transformations like rotation, scaling, and tonal adjustments. Preprocessing varies by method: [48] uses wavelet transformation with multi-frequency sampling; [47] enhances contrast and brightness during inference. CASDD [28] employs a Generative Adversarial Network (GAN) to generate synthetic images, boosting dataset variety and robustness.

Deep supervision is widely adopted: [1,2] apply loss to the original mask, while [3,4] use edge masks with boundary loss. Training splits typically allocate 60–80% of data to training, with the rest for validation and testing. Five-fold cross-validation [91] is used in some studies, rotating validation sets across five cycles. Pretrained backbones (e.g., ImageNet, ADE200) are fine-tuned on study-specific datasets. NVIDIA GeForce RTX 3090 (24 GB VRAM) is the most used GPU, followed by Tesla V100 (16 GB). See Appendix F, Table A5, for details.

### 4.6. Training of Architectures

Most SS models use the Cross-Entropy (CE) loss function [92] for pixel-level evaluation, as it penalizes small errors heavily, accelerating optimization [31]. Methods [30,39,42] rely solely on CE, while others create hybrid loss functions: Refs. [31,36,38] combine CE with DSC (weighted) for small region emphasis; Refs. [18,33,34] add the Structural Similarity Index Measure (SSIM) or Boundary Loss (BL) for edge focus; Refs. [29,35] use mIoU alone, with [33] blending it with CE for global overlap.

Deep supervision adds auxiliary losses in intermediate layers to guide hierarchical learning [29,31,33]. In [27], it follows signal filtering, comparing features to BL. Hyperparameters include learning rates of 10^−5^ to 10^−3^ and batch sizes of 2–20, with the Adaptative Moment Estimation (ADAM) optimizer [93] used in 70% of studies. See Appendix G, Table A6, for details.

## 5. Study Comparison

### 5.1. Application Domains and Dataset Availability

LCI SS methods support diverse applications, primarily aiding human decision-making in diagnostics and risk detection. Medical diagnostics dominate, leveraging CT, MRI, and ultrasound for tasks like artery segmentation to detect cardiovascular issues. Other applications include surface defect analysis and smoke/fire detection.

Datasets focus on single-region segmentation, with MT (magnetic tiles surface defects) and ISIC (skin lesions) being the most popular public datasets. Smoke detection datasets are scarce, with only the smoke semantic segmentation (SSS) dataset noted. Four studies created custom datasets across medical, scene understanding, mineral exploration, surface defect, and smoke categories, each with one dataset, highlighting data scarcity (RQ8).

### 5.2. Methodology and Design of the Reviewed Studies

CNN and hybrid methods adapt established architectures—UNet, DeepLab, and Segformer—to optimize LCI SS. UNet dominates, used in 87% of studies, reflecting its prevalence in medical applications, the most common domain reviewed. These DL methods enhance inference accuracy by refining base architectures with strategies that integrate local and global feature capture and fusion, often incorporating attention mechanisms to filter spatial and channel features.

Hybrid architectures combine CNNs, ViTs, and MLPs to leverage their complementary strengths. CNNs excel at local feature extraction with a high inductive bias [94], while ViTs and MLPs capture global context, offering greater implementation flexibility.

No architectural distinctions exist between single- and multi-region segmentation in either CNN or hybrid methods. The focus remains on modular techniques, such as attention and multi-scale designs, to address LCI SS challenges effectively.

### 5.3. Training and Implementation of the Reviewed Methods

Per [95], non-pretrained CNNs need ~10,000 samples for optimal crack segmentation performance without augmentation. LCI datasets face challenges: limited availability, sparse annotations, class imbalance, variable scales, and low resolution [96,97]. Data augmentation (e.g., 750 to 2400 samples [98]) and pretraining improve robustness and DSC (>80%). Hybrid methods benefit most from augmentation, as ViTs require large datasets due to low inductive bias.

Hybrid loss functions dominate, with CE leading for convergence and class imbalance handling. Five-fold cross-validation aids small datasets (<20 samples). GPU needs vary by model size: UNet (28 M parameters) requires 4 GB VRAM, UNETR (133 M) needs 24 GB [99]. Most reviewed methods (<45 M parameters) use 6 GB VRAM; RNightSeg [32] and FDR-TransUNet [41] (>100 M) require 24 GB (RQ5, RQ6).

### 5.4. Performance of the Reviewed Methods

Inference accuracy, measured primarily by mIoU and DSC, is the key evaluation metric for the reviewed methods. All 25 methods surpass their baseline architectures, achieving mIoU and DSC values above 80% in most cases, demonstrating robust LCI segmentation performance.

**Figure 8:**
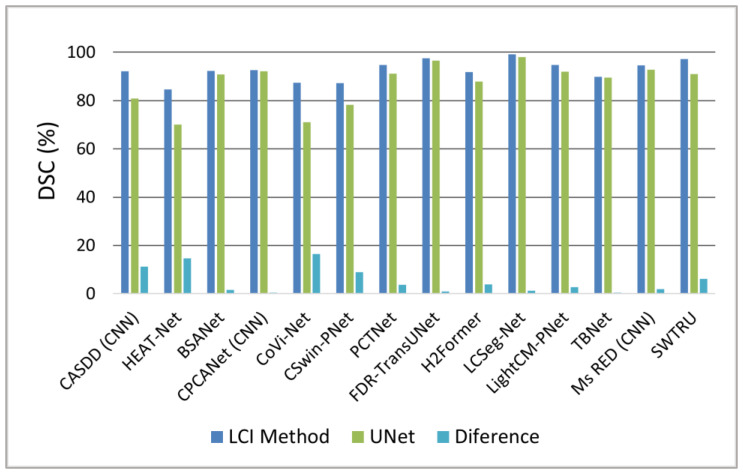
Bar chart comparing chart comparing Dice Similarity Coefficient (DSC) of reviewed methods to UNet, based on the best-performing dataset for each method.

Figure 8 illustrates performance against UNet: CoVi-Net [39] exceeds UNet by ~15% DSC, excelling at fine corneal vessel segmentation, while TBNet [45] improves by <1%, targeting corneal endothelium cells. Both highlight strengths in fine-structure detection.

**Figure 9:**
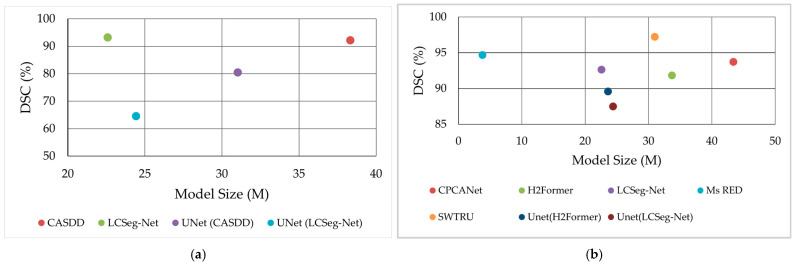
Dice Similarity Coefficient (DSC) of reviewed methods on (**a**) surface defect (MT, NEU, RSDD) and (**b**) skin lesion (ISIC, PH2) datasets, plotted against model size. Datasets within each category share similar LCI characteristics.

Figure 9 compares performance across public datasets: Figure 9a for surface defect datasets (MT, NEU, RSDD) shows LCSeg-Net [27] achieving DSC > 90% with <25 M parameters and Figure 9b for skin lesion datasets (ISIC, PH2) reveals SWTRU [47] surpassing 95% DSC, with Ms RED [46] being competitive at <10 M parameters despite a smaller model size.

**Figure 10:**
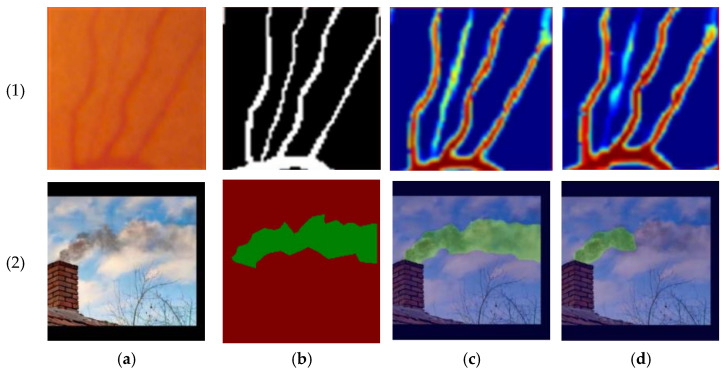
Qualitative results. (1) Results presented in [42]; (2) results presented in [35]. (**a**) Original Image; (**b**) ground truth; (**c**) LCI method; and (**d**) baseline (**Top**: UNet; **Bottom**: SegFormer).

Figure 10 presents qualitative comparisons: [42] (fine structures) and [35] (robust structures) outperform their baselines (UNet and Segformer, respectively), showing sharper LCI segmentation.

Two outliers underperform: TBNet [45] achieves 63.9% mIoU due to limited, imbalanced data; RNightSeg [32] scores 57.9% mIoU, impacted by complex night scenes with light flares and fine details. Model size typically increases over baselines, with added modules raising parameter counts, computational complexity, and training time. Inference time, reported in nine studies, varies with hardware: LCSeg-Net [27] achieves 40–70 fps (real-time), while most others average < 1 fps, reflecting GPU diversity (e.g., RTX 3090, Tesla V100).

## 6. Discussion

This review’s methodology, adapted from [21], ensures a systematic, efficient analysis by integrating tools for study selection, quality assessment, and data synthesis. This structured approach enhances rigor, transparency, and reproducibility, distinguishing it from narrative reviews like [100] that lack standardization. The quality assessment (Appendix C) proved critical, with criterion 1.3—requiring explicit focus on LCI segmentation—acting as the most effective filter for ensuring relevance and credibility.

DL methods outperform traditional techniques in LCI segmentation. For instance, K-means and Gaussian Mixture Models (GMMs) achieve < 70% accuracy in breast lesion segmentation [101], while thresholding maximum principal strain maps yields < 50% DSC in crack segmentation [102]. Reviewed DL methods, mostly hybrid, leverage CNNs, ViTs, and MLPs: CNNs capture local features with high inductive bias, ViTs excel at contextual understanding, and MLPs model nonlinear feature relationships, yielding DSC and mIoU > 80% (Section 5.4).

Architectural designs target LCI challenges universally, using techniques like attention mechanisms and multi-scale fusion (Section 4). Methods like PCTNet [30] and GT-DLA-dsHFF [42] excel at fine structures (e.g., vessels, cracks), yet no distinction exists between single- and multi-region segmentation designs. This flexibility, as shown in [103], allows single-region datasets to support multi-region extraction via probabilistic modules and trainable classifiers, suggesting adaptability across tasks.

Most methods use non-pretrained models, despite LCI dataset scarcity (Section 5.3). Pretrained models could enhance performance: LCSeg-Net [27] and PCTNet [30] leverage ResNet-34 pretrained on ImageNet-10K and ADE20K, though these datasets feature balanced contrast. MedSAM [104], pretrained on 1.5 million medical LCI images, offers a promising alternative for automated diagnostics, addressing data limitations (RQ8).

Ethical, social, and legal implications of AI-assisted decision-making are significant. Human–machine collaboration is growing [105], raising concerns about accountability for errors and penalty allocation [106]. Future LCI SS research must prioritize transparency to build trust in automation applications (Section 8).

## 7. Conclusions

This systematic review comprehensively assessed recent DL advancements for the SS of LCIs, offering a detailed evaluation of their strengths and limitations across diverse applications. Hybrid architectures integrating CNNs and ViTs, enhanced by attention mechanisms, have proven exceptionally effective at tackling the inherent challenges of LCI segmentation, such as diffuse edges and tonal similarities that obscure object boundaries. These methods leverage CNNs’ ability to extract local spatial details and ViTs’ capacity to capture global contextual relationships, resulting in superior performance over traditional approaches. Multi-scale processing and ERF expansion further boost accuracy in complex scenes (Section 4), enabling models to adapt to varying object scales and intricate backgrounds often encountered in LCI scenarios. Among the standout methods, SWTRU achieves top-tier performance in medical segmentation, excelling at delineating fine anatomical structures critical for diagnostics; Ms RED stands out with the smallest model size, offering an efficient solution without compromising accuracy; and LCSeg-Net enables real-time inference, demonstrating robustness across multiple domains with processing speeds suitable for practical deployment (Section 5.4). These examples highlight the diversity of innovations, from high-precision medical applications to resource-efficient and time-sensitive industrial or autonomous systems.

Despite significant progress in accuracy and robustness, several persistent challenges underscore the need for further development. The scarcity of diverse, large-scale LCI datasets remains a critical bottleneck, limiting model training and generalization across varied real-world conditions (RQ8). This data scarcity is particularly pronounced in domains like smoke detection and mineral exploration, where public datasets are rare, forcing reliance on small or custom datasets that may not fully represent operational complexities. Additionally, the high computational demands of transformer-based models pose a barrier, requiring substantial processing power and memory that may not be feasible in resource-constrained environments, such as edge devices or mobile platforms. The limited availability of real-time solutions exacerbates this issue, as most methods struggle to achieve the speed necessary for applications like autonomous navigation or industrial monitoring, where immediate decision-making is essential. These challenges collectively hinder the scalability and accessibility of LCI SS, particularly in settings where computational resources or annotated data are sparse.

DL significantly enhances LCI SS across a wide range of sensor-driven domains, including medical imaging, autonomous driving, industrial inspection, and remote sensing, transforming how intelligent systems interpret challenging visual data. In medical imaging, it enables the precise detection of subtle tissue boundaries, supporting earlier and more accurate diagnoses. In autonomous driving, it improves scene understanding under poor lighting, enhancing safety and reliability. Industrial inspection benefits from better defect detection on uniform surfaces, ensuring quality control, while remote sensing gains from improved environmental analysis despite shadows or tonal overlap. Yet, to fully unlock this potential, optimizations are critical in three key areas: computational efficiency, data availability, and model interpretability (Section 6). Efficiency improvements could democratize access to these technologies, making them viable for smaller organizations or low-power devices. Enhanced data availability, through expanded datasets or synthetic generation, would strengthen model robustness, reducing overfitting and improving adaptability. Greater interpretability would build trust, especially in safety-critical applications, by clarifying how models reach their segmentation decisions. Together, these advancements promise to bridge current gaps, paving the way for broader adoption and impact in automation and robotics.

## 8. Future Perspectives

This review identifies key research directions to advance LCI SS:

- Computational Efficiency: Developing lightweight, energy-efficient transformer architectures is essential for real-time deployment. Techniques like quantization, knowledge distillation, and pruning can cut computational costs without sacrificing performance (RQ7).
- Dataset Expansion and Augmentation: Limited high-quality, annotated LCI datasets hinder progress (Section 5.3). Future efforts should create diverse, large-scale datasets spanning multiple domains. Advanced augmentation, such as synthetic image generation, can address data scarcity, including enhancing image sharpness to aid segmentation (RQ8).
- Self-Supervised and Few-Shot Learning: Reducing reliance on labeled data, self-supervised and few-shot learning can improve generalization with minimal supervision, such as correlating pixels across classes for enhanced segmentation (RQ7).
- Real-Time and Mobile Deployment: Enhancing real-time performance on resource-limited devices is vital. Efficient baseline frameworks and mobile-optimized architectures can balance performance and deployability (RQ7).
- Integration of Multimodal Sensor Data: Fusing LCI with modalities like LiDAR, thermal, or hyperspectral data can improve accuracy in challenging conditions. Developing models to leverage multimodal inputs is a priority (RQ7).
- Ethics, Interpretability, and Explainability: As DL influences safety-critical decisions, transparency and trust are paramount. Explainable AI techniques must illuminate model decisions, especially in medical and autonomous navigation contexts (Section 6).

Addressing these gaps will drive LCI SS forward, enhancing performance and enabling broader sensor-driven applications.

## Figures and Tables

**Table 1:** Summary of the key characteristics of the reviewed methods.

Ref.	Name	Type	Mechanism to Enhance ERF	Parameters (M)	Maximum Performance (%)	Dataset	Highlights	Limitations
	Surface Defect							
[28]	CASDD	CNN	AC	38.34	DSC 92.1	NEU/RCD/OPDD	Improves adaptability to data variability.	Poor defect detection with an aspect ratio of 0.4 or lower.
[29]	EMRANET	CNN	BAM+RA	NR	mIoU 87.36	DAGM 2007/MT/AITEX/RSDD	Optimizes the extraction and fusion of global features.	Poor defect detection with a low aspect ratio.
[30]	PCTNet	CNN+ViT	AC+MHSA	30.05	mIoU 90.53, DSC 94.8	Crack R	Expands receptive field for lower-level features while limiting it for higher-level features.	High computational cost.
[31]	STCNet II	CNN	ASPP	NR	mIoU 87.07	Own dataset	Improves accuracy by enlarging the ERF while maintaining image resolution.	Only captures objects between 0.1 and 0.3 mm in width.
	Scene Understanding							
[18]	NR	CNN	AC+CA	NR	mPa 93.24, mIoU 90.82	Own dataset	Suppresses background information interference.	Fine details are not captured.
[32]	RNightSeg	CNN+ViT+MLP	MHSA	104.16	mIoU 57.91	BDD100K-Night/ NightCity+	Handles over- or underexposure caused by uneven lighting.	High computational cost, not real-time capable.
	Mineral Exploration							
[33]	FAM-CRFSN	CNN	AC	24.57	mIoU 85.77, mPa 92.12	Own dataset	Optimizes the extraction of base architecture features.	High computational cost.
	Remote Sensing							
[34]	GVANet	CNN+ViT+MLP	BAM+MLP	28.59	mIoU 87.6, DSC 92.82	ISPRS-Vaihingen/ ISPRS- Postdam	Enables multiview expansion of single-view information and cross-level information interaction.	Poor edge detection.
	Smoke Detection							
[27]	LCSeg-Net	CNN+MLP	MLP	22.6	DSC 95.82, mIoU 92.02	SSD	Reduces noise in edge inference by applying filters and using uncertainty models during feature fusion.	High dependency on label resolution.
[35]	SmokeSeger	CNN+ViT+MLP	DwC+MHSA+MLP	34	mIoU 91.6	USS/SSS	Optimizes global and local feature capture and fusion.	Not real-time capable for practical applications.
	Medical							
[36]	HEAT-Net	CNN+ViT	DA+MHSA	NR	DSC 94	BUSI/DDTI/ TN3k/CAMUS	Reduces localization errors caused by structural and pixel intensity similarity.	Not real-time.
[37]	BSANet	CNN+ViT	DwC+MHSA	14.15	DSC 96.48	RVSC/SCD/ SunnyBrook	Improves adaptation to scale and shape variations.	Application-specific.
[38]	CPCANet	CNN	CPCA	43.43	DSC 93.7	ACDC/ISIC 2016/PH2/Synapse/EM	Modifies the attention module of the base model to make it more efficient and lightweight.	Does not support datasets of different sizes than the one used in the study.
[39]	CoVi-Net	CNN+ViT	DS+MHSA	22.99	Acc 97.1	DRIVE/CHASEDB1/STARE	Incorporates local and global features in a transformer.	Overfitting during training; high computational cost.
[40]	CSwin-PNet	CNN+ViT	SE+MHSA	NR	DSC 87.25	UDIAT	Optimizes global and local feature fusion between the encoder and decoder.	Poor classification of diffuse areas.
[41]	FDR-TransUNet	CNN+ViT	MHSA	101	mIoU 90, DSC 97.5	COVID-19 Radiography	Proposes an encoder adjusted to depth, retaining more local features.	Does not support data variability; high computational cost.
[42]	GT-DLA-dsHFF	CNN+ViT	AC+SE+MHSA	26.08	DSC 86.5	DRIVE/STARE/CHASE_DB1/HRF	Applies local and global attention modules in parallel, consolidating edge and fine detail detection.	High computational cost.
[43]	H2Former	CNN+ViT	MHSA+SE	33.71	DSC 91.8, mIoU 86.29	IDRiD/KVASIR SEG/SKIN LESION	Implements attention modules at various depths, improving feature representation capability.	Fails to capture diffuse areas and small objects.
[44]	LightCM-PNet	CNN+MLP	MLP+DS	28.78	DSC 92.42	Own dataset	Real-time inference; enhances channel information exchange and context information perception.	Poor segmentation of diffuse areas.
[45]	TBNet	CNN+ViT	MHSA	14.45	DSC 80.26, mIoU 67.14	TM-EM3000/ ALIZARINE/ SP-3000	Optimizes global and local feature capture and fusion.	Over-segmentation in regions with few pixels.
[46]	Ms RED	CNN	DC+CA+SA	3.8	DSC 94.65, mIoU 90.14	ISIC 2016-2017-2018/PH^2	Reduces the number of parameters in the base model, requiring fewer labeled data for training.	Focuses mainly on local features.
[47]	SWTRU	CNN+ViT	MHSA	31	DSC 97.2, mIoU 94.9	CHAOS/ISIC 2018/LGG	Efficiently captures global features.	Large parameter count compared to baselines; high convergence time during training.
[48]	TD-Net	CNN+ViT	MHSA+DeC	NR	DSC 91.22	NIH/MSD	Improves inference of diffuse edges and irregular shapes.	Requires a large dataset for training.
[49]	PPL	CNN	DC	NR	DSC 95.76, mIoU 92.23	DCA/XCA	Progressively builds context, inference, and boundary perception.	Limited generalization to specific application types.
[50]	U-NTCA	CNN +ViT	MHSA	NR	DSC 86.42, Acc 97.78	Own dataset	Generate low-level positional and morphological features that are transmitted to the upper layers to facilitate multi-scale feature fusion.	It does not consider edge constraints to address incorrectly connected cells or cells with broken edges.

NR: Not Reported; metrics reflect peak performance on primary datasets.

## Data Availability

Data are contained within the article.

## Abbreviations

The following abbreviations, listed in alphabetical order, are used in this manuscript:

ADAM	Adaptative Moment Estimation
AI	Artificial Intelligence
A-MLP	Axial MLP Attention
AC	Atrous Convolution
AIAB	Aggregation Inhibition Activation Block
ASPP	Atrous Spatial Pyramid Pooling
BAM	Bottleneck Attention Mechanism
BASE	Bielefeld Academic Search Engine
BCEn	Brightness and Contrast Enhancement
BL	Boundary Loss
BSANet	Boundary-aware and Scale-Aggregation Network
CA	Channel Attention
CAMUS	Cardiac Acquisitions for Multi-structure Ultrasound Segmentation
CASDD	Complementary Adversarial Network-Driven Surface Defect Detection
CBAM	Convolutional Block Attention Mechanism
CE	Cross Entropy
CED	Canny Edge Detection
CFD	Crack Forest Dataset
C-MLP	Channel MLP Attention
CNNs	Convolutional Neural Networks
CPCA	Channel Prior Convolutional Attention
CPCANet	Channel Prior Convolutional Attention Network
CSwin-PNet	CNN-Swin Transformer Pyramid Network
CT	Computed Tomography
CV	Computer Vision
CVC-ClinicDB	Computer Vision Center Clinic Database
FAM-CRFSN	Fuse Attention Mechanism’s Coal Rock Full-Scale Network
DA	Dual Attention
DCs	Dilated Convolutions
DeC	Deformable Convolution
DL	Deep Learning
DRIVE	Digital Retinal Images for Vessel Extraction
DS	Depth Separable Convolution
DSC	Dice Similarity Coefficient
DwC	Depthwise Convolution
EMRA-Net	Edge and Multi-scale Reverse Attention Network
ERF	Effective Receptive Field
FAM-CRFSN	Fuse Attention Mechanism’s Coal Rock Full-Scale Network
FDR-TransUnet	Feature double reuse Transformer Unet
FL	Focal Loss
GANs	Generative Adversarial Networks
GMM	Gaussian Mixture Models
GPU	Graphic Processing Unit
GT-DLA-dsHFF	Global Transformer and Dual Local Attention Network via Deep-Shallow Hierarchical Feature Fusion
GVANet	Grouped Multiview Aggregation Network
HEAT-Net	Hybrid Enhanced Attention Transformer
H2Former	Hierarchical Hybrid Vision Transformer
ISIC	International Skin Imaging Collaboration
ISPRS	International Society for Photogrammetry and Remote Sensing
JCR	Journal Citation Reports
LCI	Low-Contrast Images
LCSeg-Net	Low-Contrast Segmentation Network
LF	Loss Function
LI	Linear Interpolation
MC	Multi-Cross Attention
MF	Median Filter
MHSA	Multi Head Self Attention
mIoU	mean Intersection Over Union
MLP	Multi-Layer Perceptron
mPa	Mean Pixel Accuracy
MRI	Magnetic Resonance Imaging
Ms RED	Multi-scale Residual Encoding and Decoding Network
MT	Magnetic Tile
PCTNet	Pixel Crack Transformer Network
PICOC	Population/Problem, Intervention, Comparison, Outcome, Context
PPL	Progressive Perception Learning
PwC	Pointwise Convolution
RA	Reverse Attention
RQ	Research Question
RNightSeg	Retinex Night Segmentation
RMSProp	Root Mean Square Propagation
SA	Spatial Attention
SE	Squeeze-and-Excitation
SGD	Stochastic Gradient Descent
SPA	Spatial Pyramid Attention
SPP	Spatial Pyramid Pooling
SS	Semantic Segmentation
SSIM	Structural Similarity Index Measure
STCNet II	Slab Track Crack Network II
SJR	SCImago Journal Rank
SWTRU	Star-shaped Window Transformer Reinforced U-Net
TBNet	Transformer-embedded Boundary Perception Network
TD-Net	Trans-Deformer Network
WD	Wavelet Decomposition
ViTs	Visual Transformers

## Appendix A. PICOC Criteria

**Table A1:** PICOC criteria implemented in the present review.

Attributes	Keywords	Related
Population	LCI Datasets	Blurry Images, Diffuse Images, Poor Light Scenes
Intervention	SS Architectures with attention mechanism	Channel Attention, Spatial Attention
	SS Architecture with skip and dense connections	Unet, DenseNet, HRNet
	SS Architectures with atrous convolutions	Dilatated Convolution, ASPP, Deeplab, PSPNet
	SS Hibrid Architecture	Unext, Swin-Unet
Comparison	SS CNN-pure architectures	FCN, AlexNet, VGG
	SS Transformer-pure architectures	ViT, EfficientViT
Outcome	Inference Accuracy	mIoU, DSC
	Inference Speed	FLOPs, Latency
Context	Diseases Diagnosis	CT Images Analysis
	Autonomous Vehicles	Scene SS
	Remote Sensing	Cloud and Snow SS
	Surface Defect Detection	Surface Anomaly Detection
	Mineral Exploration	Mineral Prospecting
	Smoke Detection	Initial Fire Detection

## Appendix B. Inclusion and Exclusion Criteria

**Table A2:** Inclusion and exclusion criteria applied to publications found in the selected repositories and search engines.

Criteria Type	Inclusion	Exclusion
Period	Publications from 2022 onwards	Publications before 2022
Language	Publications in English	Publications other than English Language
Type of Source	Peer-reviewed journal articles	Conference proceedings, technical reports, books, dissertations, and non-peer-reviewed works
Impact Source	Journals ranked in Q1 (based on JCR or SJR metrics)	Journals ranked in Q2–Q4 (or not indexed in JCR or SJR)
Accessibility	Studies available in BASE, Google Scholar and Refseek.	Studies not available in BASE, Google Scholar and Refseek.
Research Focus	DL techniques	Classical machine learning techniques
	Supervised learning approaches	Unsupervised or self-supervised learning approaches
	Studies focusing on image-based semantic segmentation	Studies focusing on video segmentation or synthetic data or other computer vision task

## Appendix C. Quality Assessment Checklist

**Table A3:** Instrument used to evaluate the rigor, relevance, presentation of results, and credibility of the selected publications after applying the inclusion and exclusion criteria.

	Judgement Criteria	Options
1. Scope and Objectives	1.1. Is the review scope clearly defined?	Yes: The review scope is clearly defined, focusing on DL methods for low-contrast images.
		No: The scope is unclear, other or not defined.
	1.2. Are the research questions aligned with the objectives?	Yes: The research questions align with the defined objectives and address key issues.
		No: The questions are not aligned with the objectives or are missing.
	1.3. Does the objective of the DL methods explicitly focus on segmenting datasets composed of low-contrast images of real-world applications?	Yes: The objective explicitly addresses the segmentation of low-contrast images using DL methods for real-world applications.
		No: The objective does not explicitly address the segmentation of datasets composed of low-contrast images using DL methods or focuses on other topics related to datasets composed of low-contrast images for real-world applications.
2. Methodology	2.1. Are the method described in sufficient detail?	Yes: The methods are well-described, highlighting key components and relevance to low-contrast images.
		No: The architectures are not described or descriptions are incomplete.
	2.2. Are the methods compared with deep learning state-of-the-art models?	Yes: The methods are adequately compared with state-of-the-art models, with results clearly contextualized.
		No: No comparison is made.
	2.3 Are the selected datasets suitable for the supervised training of the models?	Yes: The selected datasets are suitable for the supervised training of the models.
		No: The selected datasets are not suitable for the supervised training of the models.
3. Performance Evaluation	3.1. Does the review discuss performance?	Yes: Performance in low-contrast scenarios is clearly discussed with relevant metrics and analysis.
		No: This aspect is not addressed.
	3.2. Are standard semantic segmentation indexes used to evaluate the performance of the method?	Yes: Standard indexes are used for evaluation.
		No: Standard indexes are not used for evaluation.
4. Challenges	4.1. Are the proposed future directions related to the method?	Yes: Clear and relevant future directions are proposed, considering current challenges.
		No: No future directions are proposed or are vague.
	4.2. Are the limitations of the method discussed?	Yes: Limitations are discussed.
		No: No limitations are discussed or are vague.
Verdict	High Quality	All judgments are Yes.
	Low Quality	At least one of the responses is No.

## Appendix D. Publications Excluded After Quality Assessment

**Table A4:** Classification of the excluded publications based on the judgment criteria number they did not meet from quality assessment checklist.

Publications	Judgment Criteria Number
[107,108,109,110,111,112,113,114,115,116,117,118,119,120,121,122,123,124,125,126,127,128,129,130,131,132,133,134,135,136,137,138,139,140,141,142,143,144,145,146,147]	1.3
[148]	2.1
[149]	3.2
[15,150,151,152,153,154,155,156,157,158,159,160,161,162,163]	4.1
[164,165,166,167,168,169,170,171,172,173,174,175,176,177,178,179,180,181,182,183,184]	4.2

## Appendix E. Public Dataset for Low-Contrast Semantic Segmentation

**Table A5:** Characteristics of some public datasets used by the methods in the reviewed publications.

Dataset	Ref.	Utilized by	Segmentation Performance (%)	Image Resolution	Image Count	Segmented Object
AITEX	[56]	[29]	82.56 (mIoU)	4096 × 256	245	Textile defect
MT	[57]	[29]	83.75 (mIoU)	219 × 264	1344	Magnetic tile surface defects
TN3K	[58]	[36]	89.5 (DSC)	421 × 345	3493	Thyroid nodules
CAMUS	[59]	[36]	92.2 (DSC)	512 × 512	2000	Breast lesion
SCD	[60]	[37]	92.39 (DSC)	512 × 512	805	Left ventricle
ISIC 2016	[61]	[38]	93.7 (DSC)	1504 × 1129	1279	Skin Lesion
CVC-ClinicDB	[52]	[27]	95.82 (DSC)	384 × 288	612	Colon polyps
ISPRS-Potsdam	[62]	[34]	93.29 (DSC)	6000 × 6000	38	City objects
Nightcity	[63]	[32]	57.91 (mIoU)	1024 × 512	4297	City objects
DRIVE	[64]	[42]	86.5 (DSC)	565 x 684	40	Retinal Vessels

## Appendix F. Implementation Details of Reviewed Methods

**Table A6:** Key parameters for the implementation of the reviewed methods. In ‘Dataset division’, three values indicate the order training/validation/test, while two values represent training/validation. Minimum training set size refers to the number of samples used for training from the dataset with the fewest data. CVa indicates the cross-validation method, with the accompanying number representing the number of folds implemented. DAg: data augmentation; MF: Median Filter; LI: Linear Interpolation; CED: Canny Edge Detection; WD: Wavelet Decomposition; NR: Not Reported; NA: Not Applicable.

Ref.	Name	Data Preprocessing	Dataset Division (%)	Minimum Training Set Size	Nvidia GPU Model	VRAM
[28]	CASDD	Dag + GAN	80/15/5	750	GeForce RTX 2060	6
[29]	EMRANET	NA	80/20	145	GeForce GTX 1660	6
[30]	PCTNet	DAg	80/20	685	GeForce RTX 3090	24
[31]	STCNet II	DAg	80/10/10	800	GeForce RTX 3080Ti	12
[18]	NR	MF + LI	70/15/15	2205	GeForce rtx 2080 ti	11
[32]	RNightSeg	DAg	70/30	320	GeForce RTX 3090	24
[33]	FAM-CRFSN	DAg	NR	NR	GTX 1650 Ti	16
[34]	GVANet	DAg	60/40	19	GeForce RTX 2080 Ti	11
[27]	LCSeg-Net	Dag + CED	80/10/10	489	GeForce RTX 3090	24
[35]	SmokeSeger	DAg	80/20	332	Tesla V100	16
[36]	HEAT-Net	DAg	5 CVa	33	Tesla V100	16
[37]	BSANet	DAg	70/10/20	32	GeForce RTX 3090	24
[38]	CPCANet	DAg	70/10/20	630	GeForce RTX 3090	24
[39]	CoVi-Net	DAg	10 CVa	18	GeForce RTX 3090	24
[40]	CSwin-PNet	NA	60/20/20	754	GeForce RTX 3080	10
[41]	FDR-TransUNet	DAg	60/20/20	1750	GeForce RTX 3090	24
[42]	GT-DLA-dsHFF	DAg	4CVa	15	Tesla V-100	16
[43]	H2Former	DAg	70/10/20	20	GeForce RTX 3090	24
[44]	LightCM-PNet	IN	5 CVa	88	Quadro RTX6000	24
[45]	TBNet	Dag + CED	5 CVa	24	GeForce TITAN XP	12
[46]	Ms RED	DAg	80/20	80	GeForce RTX 2080Ti	11
[47]	SWTRU	EBC	70/20/10	1815	GeForce RTX 3090	24
[48]	TD-Net	WD	75/25	80	GeForce RTX3090	24
[49]	PPL	DAg	80/20	3712	Tesla V100	32
[50]	U-NTCA	NA	5 CVa	108	GeForce RTX3090	24

## Appendix G. Training Details of Reviewed Methods

**Table A7:** Key parameters for training the reviewed methods: CE: Cross Entropy; mIoU: mean Intersection over Union; DSC: Dice Similarity Index; BL: boundary oss; SSIM: Structural Similarity Index; SGD: Stochastic Gradient Descent; RMSProp: Root Mean Square Propagation; A: Applicable; NA: Not Applicable.

Ref.	Name	Loss Function	Maximum Learning Rate (10^−3^)	Batch Size	Epoch	Deep Supervision	Optimization Algorithms	Pretrained Model
[28]	CASDD	CE	0.1	NR	NR	NA	Adam	NA
[29]	EMRANET	mIoU	0.5	4	100	A	Adam	A
[30]	PCTNet	CE	0.045	2	NR	NA	Adam	A
[31]	STCNet II	CE + DSC	0.1	10	NR	A	Adam	NA
[18]	NR	CE + SSIM	0.1	16	120	NA	Adam	NA
[32]	RNightSeg	CE	0.06	16	80,000	NA	Adam	A
[33]	FAM-CRFSN	CE + mIoU + SSIM	0.1	NR	50	A	Adam	NA
[34]	GVANet	CE + DSC	0.6	NR	105	NA	Adam	A
[27]	LCSeg-Net	CE + DSC + BL	0.1	8	50	A	Adam	A
[35]	SmokeSeger	mIoU	1	12	40,000	A	SGD	A
[36]	HEAT-Net	CE + DSC	1	4	500	NA	NR	NA
[37]	BSANet	DSC	1	8	200	NA	Adam	NA
[38]	CPCANet	CE + DSC	5	33	250	NA	Adam	NA
[39]	CoVi-Net	CE	1	4	150	NA	Adam	NA
[40]	CSwin-PNet	CE + DSC	0.1	4	200	A	Adam	A
[41]	FDR-TransUNet	CE	0.3	4	100	A	Adam	NA
[42]	GT-DLA-dsHFF	CE	1	2	100	NA	Adam	NA
[43]	H2Former	CE + DSC	0.1	18	90	NA	Adam	A
[44]	LightCM-PNet	CE + DSC + BL	1	8	100	NA	Adam	NA
[45]	TBNet	CE	0.2	1	100	NA	RMSProp	NA
[46]	Ms RED	CE + DSC	1	NR	250	NA	Adam	NA
[47]	SWTRU	CE	0.4	16	200	NA	RMSProp	NA
[48]	TD-Net	CE + DSC	0.1	8	30	A	Adam	NA
[49]	PPL	BL	0.2	8	50	NA	Adam	NA
[50]	U-NTCA	CE + DSC	5	33	250	NA	Adam	NA
